# MiR-146a (rs2910164) Gene Polymorphism and Its Impact on Circulating MiR-146a Levels in Patients with Inflammatory Bowel Diseases

**DOI:** 10.1007/s10753-024-02108-0

**Published:** 2024-08-06

**Authors:** Rasha Ahmed Ghorab, Shaimaa H. Fouad, Ahmed F. Sherief, Eman M. El-Sehsah, Sara Shamloul, Sara I. Taha

**Affiliations:** 1 Department of Clinical Pathology, Faculty of Medicine, Ain-Shams University, 11591 Abbasia, Cairo, Egypt; 2 Department of Internal Medicine /Allergy and Clinical Immunology, Faculty of Medicine, Ain-Shams University, Cairo, Egypt; 3 Department of Tropical Medicine, Faculty of Medicine, Ain Shams University, Cairo, Egypt; 4 Department of Medical Microbiology and Immunology, Mansoura Faculty of Medicine, Mansoura, Egypt; 5 Department of Biochemistry and Molecular Biology, Faculty of Medicine, Ain Shams University, Cairo, Egypt

**Keywords:** activity, Crohn’s disease, inflammatory bowel disease, microRNA, polymorphism, ulcerative colitis

## Abstract

MicroRNA-146a (miR-146a) has been involved in the pathophysiology of inflammatory bowel disease (IBD). However, the precise processes are still not entirely understood. Contradictory studies suggest that miR-146a expression could be influenced by the miR-146a rs2910164 C > G polymorphism. This case–control study aimed to investigate the association of miR-146a rs2910164 C > G gene polymorphism and its impact on circulating miR-146a expression levels in Egyptian IBD patients. We included 40 IBD patients and 30 matched healthy controls. Genotyping of miR-146a rs2910164 polymorphism and assessment of miR-146a expression level were done using quantitative real-time PCR in all participants. MiR-146a rs2910164 GG genotype and the G allele were reported in 47% and 70% of the IBD patient group, respectively. And they were associated with increased IBD risk. All the IBD patients with the CC genotype (100%) and most of those with the CG genotype (66.67%) had an inactive disease, while most IBD patients with the GG genotype (73.68%) had an active disease. The miR-146a expression level was the highest with the CC genotype and the lowest with the GG genotype. Also, miR-146a expression level decreased significantly in IBD patients than controls and with disease activity. Combined detection of fecal calprotectin with miR-146a expression level improved the diagnostic sensitivity and the negative predictive value in differentiating IBD patients with active disease from those inactive. Our study identified a strong association of miR-146a rs2910164 GG genotype and G allele with IBD-increased susceptibility and activity in the Egyptian population. The miR-146a rs2910164 polymorphism can reduce miR-146a expression levels in these patients as well. Further research on a larger sample size and different ethnic populations can be the key to progress in establishing this genetic association.

## INTRODUCTION

Inflammatory bowel diseases (IBDs) are chronic recurrent inflammatory disorders of the gastrointestinal tract, including Crohn’s disease (CD) and ulcerative colitis (UC). The number of people with IBDs has been rising in recent years. It has been suggested that the complex interplay between intestinal flora, the host immune system, and environmental variables is what drives the pathogenesis of IBDs [1, 2]. Previous studies also reported that genetic factors are also important in the pathogenesis of IBDs [3–5].

MicroRNAs (miRNA) are single-stranded, small non-coding RNAs that can control the expression of numerous messenger RNAs (mRNA) at the post-transcriptional level, thus, they can regulate genes involved in the pathogenesis of autoimmune and inflammatory diseases [6, 7]. Notably, the minority of single nucleotide polymorphisms (SNPs) in miRNA loci are functional and can cause aberrant miRNA regulation by modifying their expression or maturation [8–10].

MiRNA-146a (miR-146a) has been reported to be a primary regulator of the immune response by controlling the expression of the signalling molecules involved in the nuclear factor kappa B (NF-κB) signalling pathway [9]. Numerous studies have investigated the relationship between SNPs in miR-146a with cancer and autoimmune disease susceptibility [10–12]. One of the most studied functional SNPs in miR-146a is the rs2910164 C > G SNP which is thought to result in reduced miR-146a expression levels [10]. A metanalysis study suggested that miR-146a rs2910164 SNP was significantly associated with the susceptibility to IBD [13]. Additionally, some studies reported that low expression levels of miR-146 are associated with IBD susceptibility and activity [14, 15]. However, other studies claimed that high expression levels of miR-146 are implicated in IBD pathogenesis and correlate with disease activity [16].

Having observed this extreme discrepancy between studies and given that the rs2910164 SNP in miR-146a can affect its expression levels we further explored, in the current study, miR-146a gene SNP (rs2910164) association with the disease susceptibility and activity in IBD patients and investigated the impact of this SNP on miR-146a expression levels among these patients. Also, we evaluated the clinical utility of miR-146a expression levels as a predictive biomarker for IBD activity.

## METHODOLOGY

### Sample Size Calculation

Using the PASS 15 program for sample size calculation, setting power at 80% and alpha error at 0.05, and according to Keewan and Naser, 2020 [17], miR-146a (rs2910164) CG genotype was detected at a higher incidence in IBD patients (52.6%) compared to healthy controls (21.7%). A sample size of 40 IBD cases and 30 healthy controls was needed to detect the difference between the two groups.

### Study Population

Forty patients with IBD participated in this case–control study (22 patients with UC and 18 patients with CD). The study also included thirty age- and sex-matched healthy controls. All participants were recruited from Ain Shams University Hospitals, Cairo, Egypt. Patients with colorectal cancer, concomitant systemic autoimmune disorders, or infectious colitis (e.g., *clostridium difficile*) were not included in the study.

Complete history taking, clinical examinations, appropriate radiographic studies, and laboratory investigations (such as fecal calprotectin, erythrocyte sedimentation rate (ESR), C-reactive protein (CRP), and stool analysis and culture) were performed on all IBD patients. Furthermore, histological and endoscopic analyses were used to validate the diagnosis and the exclusion criteria.

### Clinical Evaluation

The clinical evaluation of IBD activity was conducted using the partial Mayo score (≥ 2 indicates active disease) for UC which depends on scoring for rectal bleeding, stool frequency, and physician's rating [18] and the Harvey Bradshaw index (HBI) (≥ 5 indicates active disease) for CD which rates the general well-being, abdominal pain, number of liquid stools per day, abdominal mass and extra-intestinal complications [19]. Moreover, the simple endoscopic score for CD (SES-CD) was used to categorize CD patients according to severity and confirm disease activity depending on scoring for presence and size of ulcers, extent of ulcerated surface, extent of affected surface, and presence and type of any strictures. The SES-CD score is as follows: inactive (0–2), mild (3–6), moderate (7–15), severe (> 15) [20]. Meanwhile, the endoscopic Mayo score was used to categorize UC patients' severity depending on erythema, vascular pattern, friability, erosions, spontaneous bleeding, and ulceration. The endoscopic Mayo score is as follows: inactive (0), mild (1), moderate (2), and severe (3) [20]. Also, the IBD extent was assessed by the Montreal classification [21]. The clinical information of all IBD participants is shown in Table 1.

**Table 1:** Stratification of IBD Patients According to the Different Disease Activity and Extent Scores (UC n = 22, CD n = 18)

		IBD patients	
		n	%
HBI clinical activity degree (CD)	Inactive	10	55.56
	Mild	3	16.67
	Moderate	4	22.22
	Severe	1	5.56
SES-CD endoscopic activity degree (CD)	Inactive	11	61.11
	Mild	3	16.67
	Moderate	4	22.22
Montreal location (CD)	L1: Ileal	4	22.22
	L2: Colonic	6	33.33
	L3: Ileocolonic	7	38.89
	L4: Isolated upper excluded	1	5.56
Montreal Behavior (CD)	B1: Non- stricturing & non-penetrating	7	38.89
	B2: Stricturing	7	38.89
	B3: Penetrating	3	16.67
	P: Perianal	1	5.56
P. Mayo clinical activity degree (UC)	Inactive	9	40.91
	Mild	4	18.18
	Moderate	7	31.82
	Severe	2	9.09
E. Mayo endoscopic activity degree (UC)	Inactive	6	27.27
	Mild	7	31.82
	Moderate	2	9.09
	Severe	7	31.82
Montreal extent of lesion (UC)	E1: Proctitis	6	27.27
	E2: Left sided (distal to the splenic flexure)	7	31.82
	E3: Extensive (proximal to the splenic flexure)	9	40.91
Radiology CT\MRI (CD)	No ileitis	7	38.89
	Ileitis	11	61.11

*CD* Crohn’s disease, *CT* computed tomography, *E. Mayo* endoscopic Mayo, *HBI* Harvey Bradshaw index, *MRI* magnetic resonance imaging, *P. Mayo* partial Mayo score, *SES-CD* simple endoscopic score CD, *UC* ulcerative colitis

### Ethical Considerations

Every participant gave their written informed consent before the start of the studies. FMASU R108/2024 is the IRB clearance number for the study, which was authorized by the Research Ethics Committee of the Faculty of Medicine at Ain Shams University in Cairo, Egypt.

### Blood Sample Collection

Each participant in this study had eight millilitres (8 mL) of blood drawn under strict aseptic circumstances, which were then separated into four parts: the first 2 mL of blood was collected into a vacutainer tube containing 3.2% sodium citrate in order to analyze the ESR using the Westergren method [22]. The second 2 mL of blood was drawn into a gel vacutainer tube, centrifuged for 20 min at 4000 rpm, and the resulting serum was utilized to measure CRP with the Cobas 6000, c501 autoanalyzer (Roche Diagnostics, Switzerland). The final 4 mL of the blood sample was split between two EDTA vacuum tube containers, one of which underwent two centrifugation processes. Following a 10-min first centrifugation at 1600 g, a second, high-speed centrifugation at 16,000 g for 10 min was performed on the resulting plasma supernatant. The level of miR-146a expression was measured using the final plasma collected. For the miR-146a rs2910164 SNP genotyping, the remaining 2 mL of whole EDTA blood was utilized.

### Assessment of Fecal Calprotectin

The stool specimen was extracted using the CALEX® Cap (BÜHLMANN Laboratories AG, Switzerland), and the resulting supernatant was analyzed on the Cobas 6000, c501 autoanalyzer (Roche Diagnostics, Switzerland) to measure fecal calprotectin.

### Genotyping for MiR-146a C > G SNP (rs2910164)

#### DNA Extraction

Genomic DNA was isolated from whole blood by using the German QIAamp DNA blood mini kit. The extraction procedure was followed as instructed in the kit handbook. Using a nanodrop (Thermo Fisher Scientific Inc., USA), the concentration of extracted DNA was measured at 260 nm. The purity of the DNA was ascertained by comparing the absorbance at 260 and 280 nm. A ratio of around 1.8 was acceptable.

#### *Genotyping for MiR-146a* rs2910164 C > G *SNP*

From Applied Biosystems in the USA, TaqMan Universal Master Mix and TaqMan® SNP genotyping assay kit with the catalogue number, 4,351,379 and assay ID: C__15946974_10, were used for the MiR-146a C > G (rs2910164) genotyping by quantitative real-time PCR (qPCR).

The following settings were made for the 20 μL qPCR final reaction volume: 10 μL of Master Mix, 1 μL of SNP assay (20x), 7 μL of DNase-free water, and 2 μL of DNA. The following protocol was used for the amplification using the 5 Plex Rotor QPCR system (Qiagen, Germany): the step of initial activation was at 95 °C for 10 min followed by 35 cycles of denaturation step at 95 °C for 15 s followed by annealing/extension steps at 60 °C for 60 s.

### Analysis of MiR-146a Expression Level

#### Extraction of MiRNA

As directed by the manufacturer, miRNA was extracted from 200 µL of plasma using the miRNeasy Kit (Qiagen, Germany).

#### Reverse Transcription PCR (RT-PCR)

From Applied Biosystems in the USA, stem-loop RT-primer from the MicroRNA TaqMan® assay and reagents from the TaqMan® MicroRNA Reverse Transcription Kit were used to reverse transcribe the extracted miRNA to complementary DNA (cDNA) as instructed by the manufacturer.

The total volume used for the RT-reaction was 15 μL, which included 0.15 μL of dNTPs (100 mM), 1 μL of MultiScribeTM Reverse Transcriptase, 0.19 μL of RNase Inhibitor, 1.5 μL of 10 × RT Buffer, 3 μL of stem loop-RT primer (5x), 4.16 μL of nuclease-free water, and 5 μL of miRNA extract. The RT-mixture reaction was brought for incubation for 30 min at 16 °C and another 30 min at 42 °C. Subsequently, there was a 5-min stop reaction at 85 °C and a last hold at 4 °C.

#### Analysis of MiR-146a Expression Level

From Applied Biosystems in the USA, TaqMan Universal Master Mix, hsa-miR-146a (cat no. 4427975, test ID: 000468), and the U6 snRNA (cat no. 4427975, test ID: 001973) TaqMan® assay kits were used for amplification of miR-146a and U6 snRNA as an internal control for normalization.

A total of 10 μL of the Master Mix, 1 μL of microRNA assay (20x), 5 μL of cDNA, and 4 μL of RNase-free water were added together to reach the 20 uL qPCR final reaction volume.

The amplification process used a 5 Plex Rotor QPCR system (Qiagen, Germany) with the following protocol: An initial activation phase of 10 min at 95 °C followed by 45 cycles of 15 s of denaturation at 95 °C and 60 s of annealing/extension at 60 °C. Finally, after normalizing the expression of hsa-miR-146a to that of U6 snRNA, the relative quantification of each sample was determined using the delta-delta Ct ^(2−ΔΔCT)^ approach.

### Statistical Analysis

The statistical software utilized for data analysis was IBM Corp.'s Statistical Package for Social Science, version 26.0 (Armonk, NY, USA). In descriptive statistics, numbers and percentages were used for non-numerical data, and medians and interquartile ranges (IQR) were used for non-parametric numerical data. The non-parametric data between two groups were compared using the Mann–Whitney test. To assess differences between more than two groups, the Kruskal–Wallis test was employed. To investigate the association between two qualitative variables, the Chi-Square test (χ2 test) was employed. Concerning the evaluation of miR-146a C > G SNP (rs2910164), the χ2 test was used to determine the significance of deviations from Hardy–Weinberg equilibrium (HWE). The frequency of the SNP was found to be consistent with HWE across study groups (*p*-value > 0.05). Using odds ratios (OR), we determined whether the miR-146a SNP C > G (rs2910164) was associated with an increased risk of IBD or not. The overall diagnostic performance of the miR-146a expression level was evaluated using the receiver operating characteristic (ROC) curve. The *p*-value was considered statistically significant if < 0.05.

## Results

### Characteristics of IBD Patients

The current study included 40 patients with IBD and 30 healthy matched controls in age and gender. The IBD patient group included 22/40 (55%) UC patients and 18/40 (45%) CD patients. All included IBD patients had no positive family history of the disease and negative stool culture. Of them, 20/40 (50%) were in activity, and 11/40 (27.5%) were smokers. Stratification of IBD patients according to the different disease activity and extent scores is shown in Table 1.

Regarding the laboratory data of the included IBD patients, the medians (IQR) of their fecal calprotectin, CRP, and ESR levels were 150.00 (60.00—582.30) µg/g, 7.10 (3.95—11.40) mg/L, and 22.50 (10.00—50.00) mm/hour, respectively.

### Expression Levels of MicroRNA 146a

**Fig. 1:**
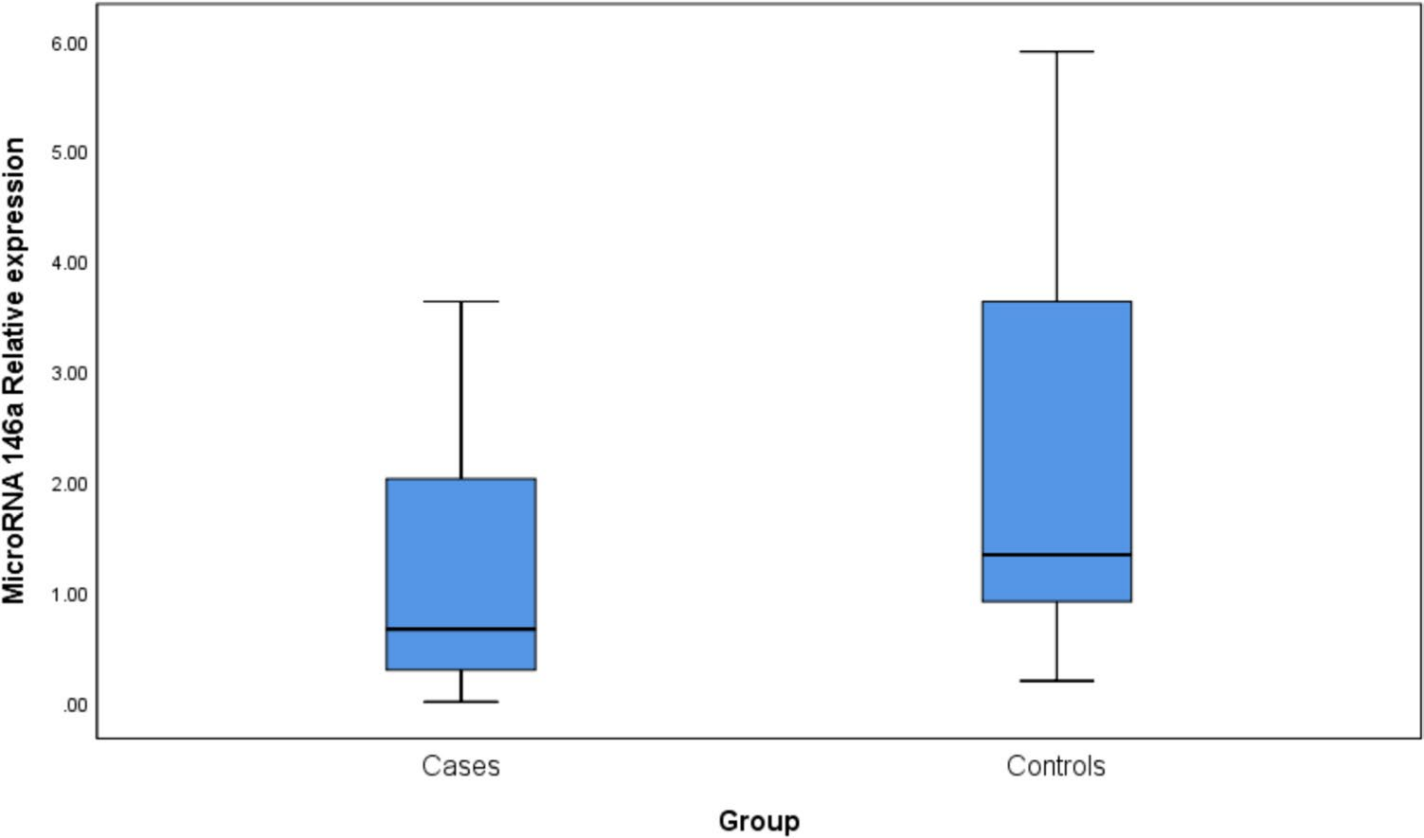
Box plots comparing miR-146a expression levels between IBD patients and controls (*p*-value = 0.013).

Comparing IBD patients to the controls, miR-146a expression levels differed significantly, being lower in IBD patients than in controls (median (IQR): 0.66 (0.29—2.03) vs 1.34 (0.91 -3.63); *p*-value = 0.013) Fig. 1.

### Distribution of MiR-146a (rs2910164) Genotypes and Alleles

There were significant differences in miR-146a (rs2910164) genotype and allele distribution between IBD patients and controls (*p*-values < 0.001). The GG genotype was the most frequent among IBD patients (19/40, 47.5%), while none of the controls expressed this genotype, with a significant (*p*-value = 0.002) odds ratio (OR) and (95% confidence interval (95%CI) of 139.28 (6.61–2932.70). On the other hand, the heterozygous CG genotype was the most frequent among the controls (18/30,60%), and 18/40 (45%) IBD patients had this genotype with a non-significant (*p*-value = 0.056) OR and 95% CI of 4 (0.96–16.61). Regarding the CC genotype, only 12/30 (40%) of controls and 3/40 (7.5%) IBD patients had this genotype. Regarding miR-146a (rs2910164) allele distribution, the C allele was the most frequent among controls (42/60, 70%), and the G allele was the most frequent among IBD patients (56/80,70%) with a significant (*p*-value < 0.001) OR and 95% CI of 5.44 (2.62–11.30) Table 2.

**Table 2:** Comparison of miR-146a (rs2910164) Genotype and Allele Distribution Between IBD Patients and Controls

MiR-146a (rs2910164)		Group				Chi-Square		OR (95% CI)	p-value
		IBD patients		Controls					
		n	%	n	%	X^2^	*p*-value		
Genotype	CC	3	7.50	12	40.00	23.450	< 0.001*	-	
	CG	18	45.00	18	60.00			4 (0.96–16.61)	0.056
	GG	19	47.50	0	0.00			139.28 (6.61–2932.70)	0.002*
Allele	C	24	30.00	42	70.00	22.015	< 0.001*	-	
	G	56	70.00	18	30.00			5.44 (2.62–11.30)	< 0.001*

^*^Bold *p*-value indicates statistical significance

### Characteristics of IBD Patients According to MiR-146a (rs2910164) Genotypes

No significant differences were observed in IBD patients’ clinical characteristics according to miR-146a (rs2910164) genotypes except for the IBD activity status (*p*-value = 0.010). All the IBD patients with the CC genotype (3/3,100%) and most IBD patients with the CG genotype (12/18, 66.67%) had inactive disease. On the other hand, most IBD patients with the GG genotype had active disease (14/19, 73.68%) Table 3.

**Table 3:** Comparison of the Clinical Characteristics of IBD Patients (n = 40) According to miR-146a (rs2910164) Genotypes

		MiR-146a (rs2910164) genotype						Chi-Square	
		CC n = 3		CG n = 18		GG n = 19			
		n	%	n	%	n	%	X^2^	*p*-value
Disease diagnosis	UC	1	33.33	9	50.00	12	63.16	1.262	0.532
	CD	2	66.67	9	50.00	7	36.84		
Activity status	Active	0	0.00	6	33.33	14	73.68	9.263	0.010*
	Inactive	3	100.00	12	66.67	5	26.32		
HBI clinical activity degree (CD)	Inactive	2	100.00	6	66.67	2	28.57	6.210	0.400
	Mild	0	0.00	2	22.22	1	14.29		
	Moderate	0	0.00	1	11.11	3	42.86		
	Severe	0	0.00	0	0.00	1	14.29		
SES-CD endoscopic activity degree (CD)	Inactive	2	100.00	7	77.78	2	28.57	5.498	0.240
	Mild	0	0.00	1	11.11	2	28.57		
	Moderate	0	0.00	1	11.11	3	42.86		
Montreal location (CD)	L1: Ileal	1	50.00	3	33.33	0	0.00	5.342	0.501
	L2: Colonic	0	0.00	3	33.33	3	42.86		
	L3: Ileocolonic	1	50.00	3	33.33	3	42.86		
	L4: Isolated upper excluded	0	0.00	0	0.00	1	14.29		
Montreal Behavior (CD)	B1: Non- stricturing & non-penetrating	1	50.00	4	44.44	2	28.57	3.320	0.768
	B2: Stricturing	1	50.00	4	44.44	2	28.57		
	B3: Penetrating	0	0.00	1	11.11	2	28.57		
	P: Perianal ds	0	0.00	0	0.00	1	14.29		
P. Mayo clinical activity degree (UC)	Inactive	1	100.00	5	55.56	3	25.00	4.957	0.549
	Mild	0	0.00	2	22.22	2	16.67		
	Moderate	0	0.00	2	22.22	5	41.67		
	Severe	0	0.00	0	0.00	2	16.67		
E. Mayo endoscopic activity degree (UC)	Inactive	1	100.00	3	33.33	2	16.67	6.417	0.378
	Mild	0	0.00	4	44.44	3	25.00		
	Moderate	0	0.00	1	11.11	1	8.33		
	Severe	0	0.00	1	11.11	6	50.00		
Montreal extent of lesion (UC)	E1: Proctitis	0	0.00	3	33.33	3	25.00	1.731	0.785
	E2: Left sided (distal to the splenic flexure)	0	0.00	3	33.33	4	33.33		
	E3: Extensive (proximal to the splenic flexure)	1	100.00	3	33.33	5	41.67		
Radiology CT\MRI (CD)	No ileitis	1	50.00	4	44.44	2	28.57	1.422	0.840
	Ileitis	1	50.00	5	55.56	5	71.43		

^*^Bold *p*-value indicates statistical significance

*CD *Crohn’s disease, *CT *computed tomography, *E. Mayo* endoscopic Mayo, *HBI* Harvey Bradshaw index, *MRI* magnetic resonance imaging, *P. Mayo *partial Mayo score, *SES-CD *simple endoscopic score CD, *UC *ulcerative colitis

Regarding the laboratory data, only miR-146a expression level (*p*-value < 0.001) and CRP levels (*p*-value < 0.041) showed significant differences according to miR-146a (rs2910164) genotypes. The expression level of miR-146a was the highest among IBD patients with the CC genotype (median (IQR): 3.63 (3.29—3.63)) and the lowest among IBD patients with the GG genotype (median (IQR): 0.34 (0.18—0.48)). On the other hand, CRP levels were the highest among IBD patients with the GG genotype (median (IQR): 9.00 (3.95 -36.95) mg/L) and the lowest among IBD patients with the CC genotype (median (IQR): 0.80 (0.75—1.65) mg/L). Table 4

**Table 4:** Comparison of Laboratory Data of the Included IBD Patients (n = 40) According to miR-146a (rs2910164) Genotypes

	MiR-146a (rs2910164) genotype												Kruskal–Wallis Test		Mann–Whitney Test		
	CC				CG				GG								
	Median	IQR			Median	IQR			Median	IQR			X^2^	P-value	CC vs CG	CC vs GG	CG vs GG
MicroRNA 146a expression (Fold change)	3.63	3.29	-	3.63	1.64	0.52	-	2.23	0.34	0.18	-	0.48	17.660	< 0.001*	0.016*	0.006*	0.001*
Fecal calprotectin (µg/g)	60.00	57.50	-	90.00	117.50	76.00	-	500.00	160.00	55.00	-	689.80	1.399	0.497			
CRP (mg/L)	0.80	0.75	-	1.65	7.10	5.00	-	11.10	9.00	3.95	-	36.95	6.372	0.041*	0.018*	0.015*	0.843
ESR (mm/hour)	7.00	5.00	-	11.50	20.00	10.00	-	48.00	40.00	15.50	-	70.00	5.433	0.066			

^*^Bold *p*-value indicates statistical significance

*CRP *c-reactive protein, *ESR *erythrocyte sedimentation rate

### Laboratory Data of IBD Patients According to Disease Activity

**Fig. 2:**
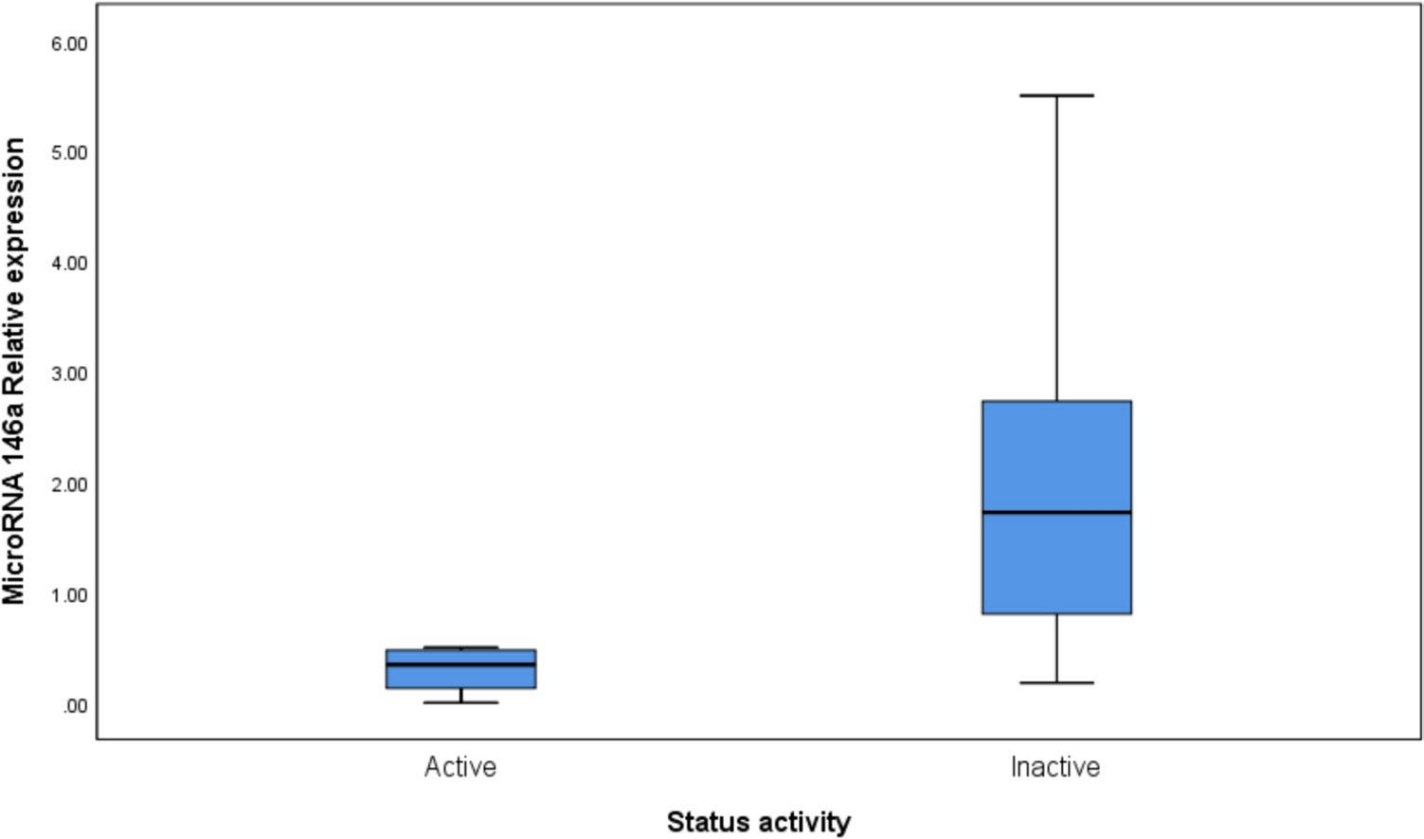
Box plots comparing miR-146a expression levels between IBD patients with active disease and those with inactive disease (*p*-value < 0.001).

There were significant differences in IBD patients’ laboratory data when compared according to IBD activity status; fecal calprotectin (*p*-value < 0.001), CRP (*p*-value = 0.009), and ESR (*p*-value < 0.001) levels significantly increased while miR-146a expression level significantly decreased (*p*-value < 0.001) in IBD patients with active disease compared to those with inactive disease. Table 5&Fig. 2.

**Table 5:** Comparison of Laboratory Data of the Included IBD Patients (n = 40) According to Disease Activity Status

	IBD activity status								Mann–Whitney Test	
	Active				Inactive					
	Median	IQR			Median	IQR			Z	*p*-value
MicroRNA 146a expression (Fold change)	0.35	0.13	-	0.48	1.73	0.81	-	2.73	3.734	< 0.001*
Fecal calprotectin (µg/g)	582.30	160.00	-	2941.50	73.00	50.00	-	102.50	3.965	< 0.001*
CRP (mg/L)	10.95	4.65	-	61.65	5.00	1.75	-	8.10	2.598	0.009*
ESR (mm/hour)	50.00	25.00	-	82.50	14.50	7.50	-	20.00	3.778	< 0.001*

^*^Bold *p*-value indicates statistical significance

*CRP *c-reactive protein, *ESR *erythrocyte sedimentation rate

### Correlations of MiR-146a Expression Level

The expression level of miR-146a showed significant negative correlations with the ESR level (r = -0.400, *p*-value = 0.010), the clinical CD activity by HBI (r = -0.531 *p*-value = 0.023), and the endoscopic CD activity by SES-CD (r = -0.531, *p*-value = 0.023). Despite the negative correlations between the expression level of miR-146a and fecal calprotectin (r = -0.104, *p*-value = 0.523), CRP levels (r = -0.236, *p*-value = 0.143), and the clinical (r = -0.418, *p*-value = 0.053) and endoscopic (r = -0.366, *p*-value = 0.094) UC activity by the Mayo score, they did not reach statistical significance.

### Diagnostic Ability of MiR-146a Expression Level

**Fig. 3:**
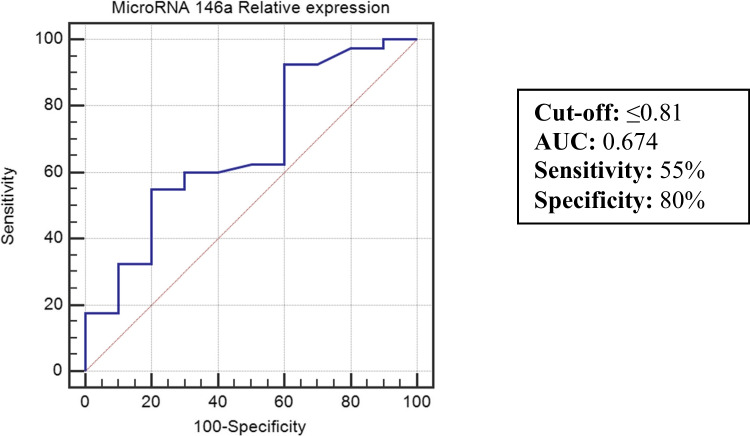
Receiver operating characteristic (ROC) curve for miR-146a for discriminating between IBD patients and controls.

Using the ROC curve analyses, miR-146a expression level at a cut-off of ≤ 0.81 was able to differentiate between IBD patients and controls with a diagnostic sensitivity of 55%, a specificity of 80%, a positive predictive value of 78.6%, a negative predictive value of 57.1%, and an accuracy of 67.4% Fig. 3.

**Fig. 4:**
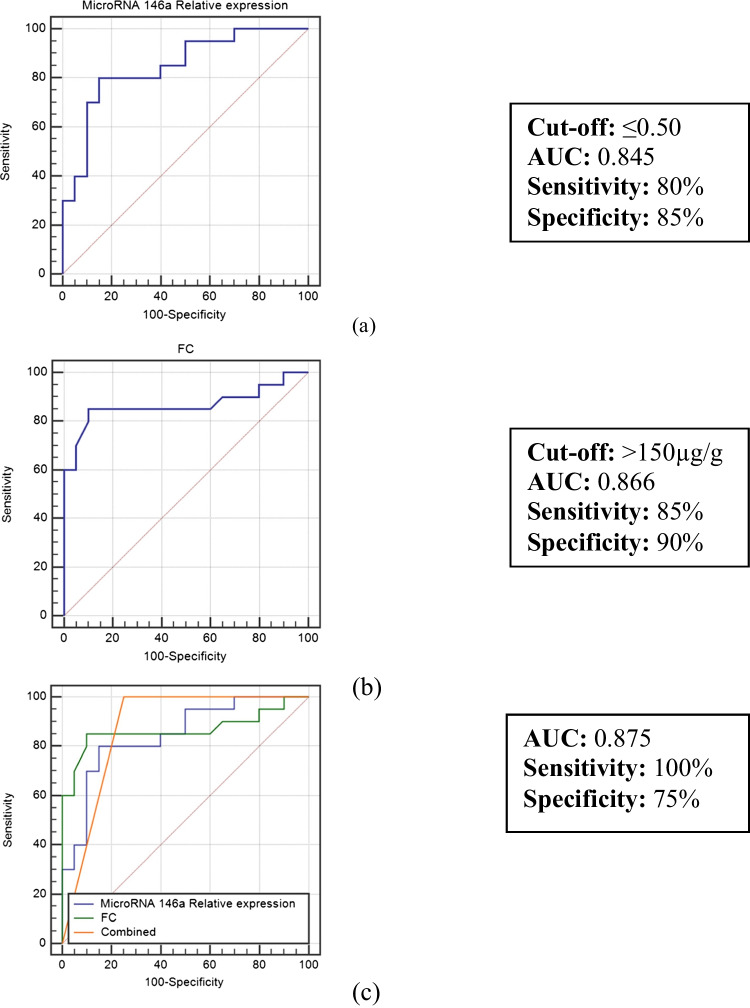
Receiver operating characteristic (ROC) curves for (**a**) miR-146a expression level, (**b**) fecal calprotectin (FC), and (**c**) combined miR-146a and FC for discriminating between IBD patients with active disease and those with inactive disease.

In assessing the ability to differentiate between IBD patients with active disease and those with inactive disease, miR-146a expression level at a cut-off of ≤ 0.50 had a diagnostic sensitivity of 80%, a specificity of 85%, a positive predictive value of 84.2%, a negative predictive value of 81%, and an accuracy of 84.5%. While fecal calprotectin at a cut-off of > 150 µg/g had a diagnostic sensitivity of 85%, a specificity of 90%, a positive predictive value of 89.5%, a negative predictive value of 85.7%, and an accuracy of 86.6%. Then, a multi-ROC curve, combining fecal calprotectin with miR-146a expression level, was constructed and showed that the diagnostic sensitivity, negative predictive value, and accuracy were increased to 100%, 100%, and 87.5%, respectively. On the other hand, the diagnostic specificity and positive predictive value decreased to 75% and 80%, respectively Fig. 4.

## DISCUSSION

This study involved several research aspects. First, the molecular analysis of miR-146a rs2910164 SNP in IBD, second, the study of the effect of miR-146a rs2910164 SNP on expression levels of miR-146a in IBD, and finally, the study of the association between miR-146a expression levels and IBD activity.

IBD is characterized by an uncontrolled inflammatory process in the intestines that requires long-term medication and/or surgical intervention. Recent studies suggest a significant link between the pathophysiology of IBD and genetic predisposition, with a large number of SNPs being related to the susceptibility and progression of IBD [23].

Previous studies have reported that miRNAs play a crucial role in the impairment of immune cell growth and/or function when the expression of miRNA is modified experimentally [24]. One of the essential regulators of both innate and adaptive immune responses is miR-146a. It was reported that miR-146a is essential for controlling the activity of the transcription factor NF-κB during inflammation; while miR-146a negatively regulates the upstream components in the NF-κB pathway (IRAK1 and TRAF6), NF-κB increases the production of miR-146a. This negative feedback loop enhances the resolution of inflammation [25]. Moreover, miR-146a also regulates toll-like receptor (TLR) and interleukin-1 (IL-1) pathways and targets key signaling proteins in the MyD88-dependent pathway, demonstrating its significance in immune responses [26]. In addition, miR-146a has a protective effect in inflammatory conditions through preventing the generation of pro-inflammatory cytokines and encouraging the polarization of M2 macrophages [27]. Previous studies proved that dysregulated miR-146a could contribute to the pathogenesis of autoimmune diseases, chronic inflammation, and malignancies [24, 28].

### MiR146a rs2910164 SNP is Associated with IBD Incidence and Activity

The current study found that miR-146a rs2910164 GG genotype and the G allele were the most predominant among the IBD patient group, and they were associated with an increased IBD risk with 139.28- and 5.44-times, respectively. On the other hand, most of the control group had the CG genotype and the C allele, and none of them had the GG genotype.

The association between the miR-146a rs2910164 SNP and IBD was investigated by several studies with different ethnic populations, however, the results were highly inconsistent. According to a study in the United States of America, Keewan and Naser, 2020 [17], reported that the miR-146a rs2910164 GC genotype was detected at a higher incidence in CD (52.6%) compared to controls (21.7%), and it was associated with upregulated inflammatory mediators [17]. Moreover, in China, Qiao et al. 2023 reported that individuals with the miR-146a rs2910164 G allele had higher serum inflammatory factor levels than individuals with the C allele [29]. A finding that supports what we have found in the current study is that the GG genotype was the most miR146a genotype associated with IBD activity.

On the other hand, a study in Greece by Gazouli et al. 2013 [30], found no significant differences in miR-146a rs2910164 genotype or allele distributions between UC and control subjects, but the CC genotype and C allele frequencies were significantly higher in CD patients than in controls. They also found that miR-146a rs2910164 did not influence the CD or UC disease characteristics [30]. Similarly, in Japan, Okubo et al. 2011 [31], reported no significant differences in miR-146a rs2910164 genotype distribution between UC patients and controls. However, in Iran, Kolahi et al. 2023 [32], reported that the miR-146a rs2910164 C allele was significantly associated with an increased risk of UC and a more severe form of the disease.

### MiR146a rs2910164 C > G SNP Affects Mir-146a Expression Level

In the current study, among the IBD patient group, miR-146a rs2910164 C > G significantly affected miR-146a expression levels; they showed the highest levels with the CC genotype and the lowest with the GG genotype. Previously, it was reported that dysregulated expression of miR-146a and their targets is associated with functional SNPs in the miR-146a sequence. It has been demonstrated that the miR-146a rs2910164 C > G reduces mature miR-146a production and silences its target genes by interfering with pre-miRNA processing [25, 33, 34]. Jazdzewski et al. 2008 [33], found that the reduction was much stronger in the presence of miR-146a rs2910164-G allele than in the C allele. A finding that suggests a direct functional effect of the miR-146a rs2910164 C > G SNP. Therefore, miR-146a rs2910164 C > G was reported to be associated with many inflammatory and pathological conditions such as cancer, severe sepsis, rheumatoid arthritis, and tuberculosis [17].

### MiR-146a Expression Level Decreases with IBD and Activity

Our study revealed that miR-146a expression level decreased significantly in IBD patients than controls, and the decrease was associated with disease activity in contrast to fecal calprotectin, CRP, and ESR, which significantly increased with disease activity. Moreover, the expression of miR-146a significantly negatively correlated with CD activity indices, but the negative correlation with UC activity indices did not reach statistical significance.

Similar to our results, Feng et al. 2020 [15], found that the expression of miR-146a was significantly lower in UC patients than in controls, and the level of miR-146a decreased significantly with the severity of UC. They also reported that miR-146a level in UC patients was negatively correlated with inflammatory markers like CRP, clinical activity index, and endoscopic index. In addition, Mirzakhani et al. 2020 [14], found that the level of miR-146a was significantly decreased in the CD patients compared to healthy controls and that CD patients with high HBI had significantly lower miR-146a. These findings could be explained by the fact that miR-146a deficiency causes a breakdown in immunological tolerance because it is commonly expressed in regulatory T cells (Tregs) and is essential for their regulatory function. Thus, the resulting uncontrolled immune response to gut microbiota can lead to gastrointestinal inflammation [35, 36]. Moreover, knocking out miR-146a in mice has been found to result in autoimmunity and increased sensitivity to lipopolysaccharides, while miR-146a activation causes concomitant reduction in the production of pro-inflammatory cytokines, suggesting its role in regulating immune responses [26].

In contrast to our results, several other studies reported the upregulation of miR-146a in the biopsies of the inflamed intestinal mucosa of IBD patients compared to the healthy controls and compared to the intact mucosa [37–39]. This finding can be explained by the effect of the local inflammatory cytokines that are involved in IBD pathogenesis, which can induce the increased expression of miR-146a in the intestinal epithelial cells as a regulatory mechanism [40].

The ethnic diversity of the study populations, the various IBD stages included in each study, and the different types of specimens used for miR-146a detection—peripheral blood, intestinal tissue biopsies, or fecal samples—could all account for these discrepancies. In addition, studies have shown a reverse pattern of miR-146a expression in the peripheral blood and colon of patients with IBD [41, 42]. Schaefer et al. 2015 [41], reported that the three distinct tissue/body fluids (saliva, blood, and colon) have different miRNA expression patterns in IBD. They found that compared to normal controls, intestinal biopsies of IBD patients showed a significant increase in miR-146a expression, but IBD patients' blood samples showed significantly lower expression of miR-146a.

Our study takes the lead in reporting that the combined detection of fecal calprotectin and miR-146a improved the diagnostic sensitivity and negative predictive value of differentiating IBD patients with active disease from those inactive. This finding should be validated by further research to confirm its clinical significance. Also, to the best of our knowledge, this study is the first to investigate the association between miR146a and IBD in the Egyptian population.

The main limitations of this research are the comparatively small sample size and the ethnic restriction to the Egyptian population, which may confine the generalizability of the results. In addition, the current study focused only on miR-146a without considering other miRNAs that may be associated with IBD. It also mainly focused on the expression of miR-146a in blood samples without considering its expression in intestinal mucosa or fecal samples. Moreover, the current research did not consider the possible impact of different treatment regimens on miR-146a expression levels. It also did not provide long-term follow-up data, which may limit the understanding of the relationship between disease progression and changes in miR-146a expression. Future research should encompass large-scale, multicenter studies with extended follow-up periods, investigate various specimen types, and consider treatment regimens.

## CONCLUSION

The results of our study demonstrated a significant association between the miR-146a rs2910164 GG genotype and G allele with increased susceptibility to and activity of IBD, as well as reduced miR-146a expression in IBD patients. Furthermore, we found that combining fecal calprotectin and miR-146a detection can enhance the sensitivity of predicting IBD activity.

## Data Availability

No datasets were generated or analysed during the current study.
